# Polylactic Acid/Bamboo Leaf Extract Electrospun Mats with Antioxidant Activity for Food Packaging Applications

**DOI:** 10.3390/antiox13121555

**Published:** 2024-12-18

**Authors:** Francesco Lopresti, Elisa Capuana, Graziella Serio, Carla Gentile, Luigi Botta

**Affiliations:** 1 Department of Engineering, University of Palermo, Viale delle Scienze, 90128 Palermo, Italy; francesco.lopresti01@unipa.it (F.L.); elisa.capuana@unipa.it (E.C.); 2 Department of Biological, Chemical and Pharmaceutical Sciences and Technologies, University of Palermo, Viale delle Scienze, 90128 Palermo, Italy; graziella.serio01@unipa.it

**Keywords:** polyphenols, electrospinning, polylactic acid (PLA), antioxidant packaging

## Abstract

This study focuses on developing an active and biodegradable packaging using electrospinning, with polylactic acid (PLA) as the matrix and bamboo leaf extract (BLE) as the antioxidant compound. The research systematically evaluates the relationship among process parameters, material properties, and structure. The electrospun membranes were produced using different BLE contents (10 wt%, 20 wt%, 30 wt%, and 40 wt%) and characterized by their morphology, mechanical properties, wettability, and antioxidant activity. Scanning electron microscopy (SEM) revealed BLE’s influence on fiber morphology, with a slight increase in diameter in PLA/BLE at 10% and 20%, attributed to higher viscosity. Conversely, PLA/BLE 30% and 40% showed a mild reduction in fiber diameter likely due to polyphenols’ capacity to enhance PLA chain mobility. Mechanical tests indicated proportional reductions in modulus, maximum stress, and strain at break, upon increasing the BLE concentration, although these parameters are still suitable for packaging applications. The decrease in modulus is attributed to polyphenol capacity to increase PLA chain mobility, while increased fragility results from embedded particles acting as local defects. Wettability tests demonstrated increased hydrophilicity with higher BLE content. Total polyphenol content, estimated through FOLIN assay, increased proportionally with incorporated BLE, impacting antioxidant properties assessed via FRAP assay.

## 1. Introduction

Within the evolving world of the food industry, packaging plays a crucial role in preserving, presenting, and attracting consumers to products [1,2]. Beyond its conventional role as a physical container, packaging is a multidimensional discipline encompassing preservation strategies [3], aesthetic considerations [4], and logistical functionality [5]. The modern food supply chain places high demands on packaging, requiring innovative solutions to address challenges ranging from microbial contamination [6,7] to environmental sustainability [8,9]. In this context, the present research lays the groundwork for exploring the potential of using state-of-the-art electrospinning technology and sustainable materials such as polylactic acid (PLA) and bamboo leaf extract (BLE) to create nanofibrous packaging with improved antioxidant properties.

The inherent nature of packaging is underscored by its role as the guardian of product integrity during the intricate journey from production to consumption. Indeed, it acts as a shield against a myriad of threats, including mechanical stresses during transport and the deleterious effects of environmental factors such as light, moisture, and oxygen [10]. Therefore, effective packaging strategies become critical to ensure food safety, quality, and appearance. The trajectory of packaging in the food industry has evolved in tandem with advances in materials, design, and consumer expectations. The modern consumer cares not only about the content of a package but also about the sustainability of its materials and the ecological footprint it leaves behind [11,12]. With growing environmental awareness, consumers are increasingly attracted to products with a smaller ecological footprint. Sustainability has emerged as a defining factor in the packaging paradigm. This paradigm shift has triggered a reevaluation of packaging materials characterized by biodegradability, recyclability, and reduced environmental impact [13,14,15,16]. In this area, bioplastics have emerged as major players [17]. These materials, derived exclusively from renewable biological sources, offer a compelling alternative to conventional plastics [18,19]. One bioplastic of note is polylactic acid (PLA), synthesized from natural monomers such as lactic acid [20]. PLA is a promising candidate for sustainable packaging due to its transparency, high solubility resistance, and favorable processing capabilities [21,22]. As a biobased material, PLA is in line with the global imperative to shift to environmentally friendly alternatives. Its biodegradability is a key feature, addressing concerns related to plastic pollution and the depletion of fossil fuel resources [23].

Despite the promise of biopolymers, challenges persist in the search for sustainable packaging solutions that balance functionality and environmental responsibility [24]. Electrospinning technology emerges as a leader in this endeavor, offering a unique platform to create biodegradable nanofibers with improved surface and structural characteristics [25,26]. Electrospinning is a technique that has transformative potential for packaging applications. Because electrospinning uses electric fields to extract nanoscale fibers from a polymer solution, it can produce materials with distinct properties. The resulting nanofibrous structure confers advantages such as higher mechanical strength, controlled permeability, and increased surface area [27]. Despite the promising applications of electrospinning in various fields at the laboratory scale in recent years, its relatively low productivity remains a key limitation for large-scale industrial implementation [28]. In response to this challenge, the development of needleless electrospinning techniques has been increasingly reported, which enhance both the productivity and quality of the resulting fibers, with the goal of facilitating industrial-scale nanofiber production, making these products more competitive than conventional packaging products [29,30].

In the food industry, antioxidants play a fundamental role in mitigating oxidation-related challenges. Among the different antioxidants, bamboo leaf extract (BLE) has been recognized for its potential applications in food preservation due to its unique composition and properties [31,32]. Bamboo leaf extract is rich in polyphenols, particularly flavonoids and phenolic acids, having strong antioxidant properties [33,34]. These compounds effectively neutralize free radicals, unstable molecules that can trigger oxidative reactions, leading to food spoilage. By eliminating these free radicals, BLE helps prevent lipid oxidation, protein degradation, and other oxidative processes that can compromise some sensory attributes and nutritional value of packaged foods [35]. In addition, BLE has demonstrated antimicrobial properties, inhibiting the growth of microorganisms and pathogens [36,37]. This dual functionality of antioxidant and antimicrobial activity makes bamboo leaf extract an ingredient that can extend the shelf life of food products. In addition, BLE has been studied for its potential synergistic effects with other natural antioxidants or preservatives, amplifying the overall effectiveness of food preservation strategies [38]. This approach is in line with the food industry’s growing trend toward natural and sustainable solutions to improve product stability [39].

The present study systematically evaluates the relationship between processing parameters, material properties, and structural characteristics of nanofibrous packaging made from PLA filled with bamboo leaf extracts. Characteristic factors investigated in this study are rheology, fiber morphology, mechanical properties, wettability, and antioxidant attributes. While intending to enhance sustainable material usage, this research contributes to active and biodegradable packaging with enhanced antioxidant properties. The ultimate goal is to provide valuable insights for applications in the food packaging industry in line with consumer, environmental, and market needs.

## 2. Materials and Methods

### 2.1. Materials

In this study, polylactic acid (PLA) (Ingeo 2002D, NatureWorks LLC, Minnetonka, MN, USA) was used as the biodegradable polymer matrix. Acetone (Ac), ethanol (EtOH), chloroform (TCM), Folin–Ciocalteu’s reagent, [2,2′-azinobis (3-ethylbenzothiazoline-6-sulfonic acid)]-diammonium salt (ABTS), 2,2-diphenyl-1-picrylhydrazyl (DPPH), 2,4,6-tripyridyl-S-triazine (TPTZ), gallic acid (GA), 6-hydroxy-2,5,7,8-tetramethylchroman-2-carboxylic acid (Trolox), potassium persulfate (K_2_S_2_O_8_), and iron (III) chloride hexahydrate (FeCl_3_ 6H_2_O) were purchased from Sigma Aldrich (Munich, Germany). A commercial formulation of bamboo leaf extract (Guardox™ BL, Handary S.A, Brussels, Belgium) was also used. According to the supplier’s data sheet, the BLE formulation contains a flavonoid concentration of at least 40 wt%, phenolic acids of at least 20 wt%, and lactones of at least 10 wt%. The product complies with Regulation (EC) No. 1334/2008 on flavorings and certain food ingredients with flavoring properties for use in and on foods.

### 2.2. Electrospinning Processing

A polymer solution was prepared for the electrospinning process. This solution consisted of 10 wt% PLA dissolved in a mixture of TCM and Ac (in a volume ratio of 2:1), according to the method described in the scientific literature [40]. To prepare the PLA/BLE suspensions, BLE particles were added to the mixture of TCM: Ac and stirred for 2 h to obtain a homogeneous suspension before adding the PLA pellets. The concentration of PLA was kept at 10% relative to the TCM:Ac mixture, while four concentrations of BLE particles were analyzed (10 wt%, 20 wt%, 30 wt%, and 40 wt% with respect to the PLA phase).

Electrospinning parameters were selected based on our previous work [41]. Specifically, a 19-gauge stainless steel needle was used, and the flow rate was set at 1 mL/h. The collector was a cylindrical rotating drum with a diameter of 10 cm, grounded, and placed at a distance of 13 cm from the needle. The PLA-based systems were processed using the following parameters to obtain a membrane approximately 70 µm thick: a high applied voltage of 18 kV, temperature maintained at 25 °C with a working humidity of 60%, the angular speed of the manifold set at 10 rpm, a flow rate of 1 mL/h, and a processing time of 180 min.

### 2.3. Morphological Analysis

The morphology of the BLE powder and the electrospun materials was assessed through scanning electron microscopy (Quanta 200 ESEM FEI, Hillsboro, OR, USA). Samples were attached to an aluminum stub using conductive carbon tape. Before SEM analysis, samples were sputter-coated with gold for 120 s (Sputtering Scancoat Six, Edwards Laboratories, Milpitas, CA, USA) to avoid electrostatic discharge during the test. The SEM was set with an accelerated voltage equal to 10 kV.

### 2.4. Particle Size and Fiber Diameter Distributions

An image processing software was used to analyze the fiber diameter and particle size distribution within the electrospun mats. SEM images of the BLE particles were analyzed with ImageJ to determine the particle size distribution. To study the diameter distribution of the fibers, a plugin for ImageJ (version 1.53a) called DiameterJ was used [42].

### 2.5. Rheological Characterization of Polymer Solutions

The rheological study of the solution was conducted using the ARES G2 instrument (TA Instrument, New Castle, DE, USA). This instrument is equipped with two 25 mm diameter plates, one fixed and the other movable. The solution was introduced onto one of the plates using a syringe while ensuring the entire plate was filled during the test. The rheological study used a frequency sweep approach with operating conditions at a T of 25 °C and imposing an interval of angular frequency between 100 and 1 rad/s to minimize solvent evaporation. The evaluation provided the value of the complex viscosity (measured in Pa s) of the polymeric solution.

### 2.6. Mechanical Properties

A 1 kN load cell was used with the Instron universal testing machine (UTM, Instron, Norwood, MA, USA, model 3365) for mechanical tensile measurements. Specimens were cut from the rotation direction of a cylinder-shaped manifold with a size of 10 × 90 mm. The thickness of each specimen (equal to 70 ± 5 µm) was evaluated before each test. Tensile tests were performed at a speed of 1 mm/min of the crosshead until failure, and the distance between the jaws of the UTM was 20 mm. Nominal stress–strain curves were used to evaluate the elastic modulus (E), tensile strength (TS), and strain at break (ε_b_) of the specimens. Seven samples were tested for each material, and the mean values of mechanical parameters and their standard deviations were reported.

### 2.7. Water Contact Angle Measurements

Static contact angle measurements were evaluated with the FTA 1000 instrument (First Ten Ångstroms, Cambridge, UK). A total of 4 ± 0.05 μL of distilled water was released from the samples. Images were acquired 10 s after deposition. At least 7 acquisitions were made for each PLA-based material.

### 2.8. BLE Solution

A weighed aliquot of the powdered bamboo leaf extract (BLE) was resuspended in an ethanol/water mixture (70/30, *v*/*v*) using a 1:100 (*w*/*v*) ratio. The sample was agitated in a vortex for 5 min, then sonicated at room temperature for 20 min, and subsequently left under continuous agitation at room temperature for a period of 1 h. Subsequently, it was centrifuged at 10,000 rpm for 10 min at 4 °C. The supernatant was recovered and stored at −20 °C until further analysis.

### 2.9. PLA Electrospun Mat Extracts

For the preparation of extracts from both control and filled electrospun mats, weighed samples were extracted using a mixture of ethanol and water (70/30, *v*/*v*) at a ratio of 1 part sample to 50 parts extraction solvent (*w*/*v*). Two samples were analyzed for each condition. Following the addition of extraction solvent, each sample underwent vortexing for 5 min and sonication at room temperature for 20 min. Subsequently, the samples were agitated at room temperature for 1 h and then centrifuged at 10,000 rpm for 10 min at 4 °C. The resulting supernatant was recovered and stored at −20 °C until further analysis.

### 2.10. Total Polyphenolic Content

The total polyphenolic content (TPC) of the BLE solution, both control and filled PLA electrospun mat extracts, and food simulants was determined using the Folin–Ciocalteu colorimetric assay [43]. Each sample was suitably diluted in distilled water, followed by the addition of Folin–Ciocalteu reagent. After a 4 min incubation period at room temperature, 10% Na_2_CO_3_ solution in water was added. The resulting mixture was then brought to the desired volume with distilled water and incubated in darkness at room temperature for 90 min. At the end of the incubation period, absorbance at 700 nm was measured. Polyphenolic content quantification was based on the GA standard, and results were expressed as gallic acid equivalent (GAE) per weight of the sample. All measurements were conducted in triplicate.

### 2.11. Antioxidant Activity

The antioxidant activity of the BLE solution, as well as both control and filled PLA mat extracts, was evaluated using three standard in-solution methods. The ABTS and DPPH assays were utilized to determine the radical-scavenging activity of the samples by assessing the decolorization of ethanol solutions containing the ABTS^+^ cationic radical or the DPPH radical, respectively [44,45]. The ABTS^+^ cationic radical was generated by the oxidation of ABTS salt with potassium persulfate and monitored at 734 nm. The absorbance of the DPPH radical solution was measured at 520 nm. Both assays were performed following established protocols [46]. The Ferric Reducing Antioxidant Power (FRAP) assay determined the reduction of the Fe^3+^-TPTZ complex to the ferrous Fe^2+^-TPTZ form, which was monitored spectrophotometrically at 595 nm [47]. The FRAP reagent was freshly prepared by combining acetate buffer (0.3 M, pH 3.6), TPTZ (10 mM in 40 mM HCl), and FeCl_3_ (20 mM). The assay was conducted in 96-well microplates according to established procedures [46]. Standard curves were generated using Trolox, a synthetic analog of vitamin E. The results from the ABTS, DPPH, and FRAP assays were expressed as mmoles of Trolox equivalents (TE) per unit weight of the sample. All measurements were carried out in triplicate.

### 2.12. Release Studies

To investigate the release of BLE antioxidant compounds from the filled electrospun mats, water/ethanol solutions were utilized as food simulants. Specifically, mixture A (ethanol/water, 50/50, *v*/*v*) served as a lipophilic food simulant, while mixture B (ethanol/water, 10/90, *v*/*v*) acted as an aqueous food simulant. All release studies were conducted at room temperature. The release tests involved immersing each electrospun sample in an appropriate volume of either mixture A or B, maintaining a weight-to-volume ratio of 1 mg/mL. At specified time intervals ranging from 0 to 53 h, the food simulant was withdrawn and replaced with a fresh food simulant. The amount of antioxidants released into the food simulant at each time point was determined by assessing the total polyphenolic content using the Folin–Ciocalteu assay, following established procedures. After the release experiments, the percentage of antioxidant compounds released from the functionalized membrane relative to the nominal content of incorporated BLE was evaluated.

### 2.13. Statistical Analysis

All statistical analyses were performed using GraphPad Prism predictive analysis software (GraphPad Software, version 8.0.2, San Diego, CA, USA). The results are expressed as mean ± standard deviation (*n* = 3) and were subjected to analysis of variance (ANOVA) at a 0.05% probability level (*p*).

## 3. Results and Discussion

### 3.1. Rheological Behavior of Polymer Solutions

**Figure 1:**
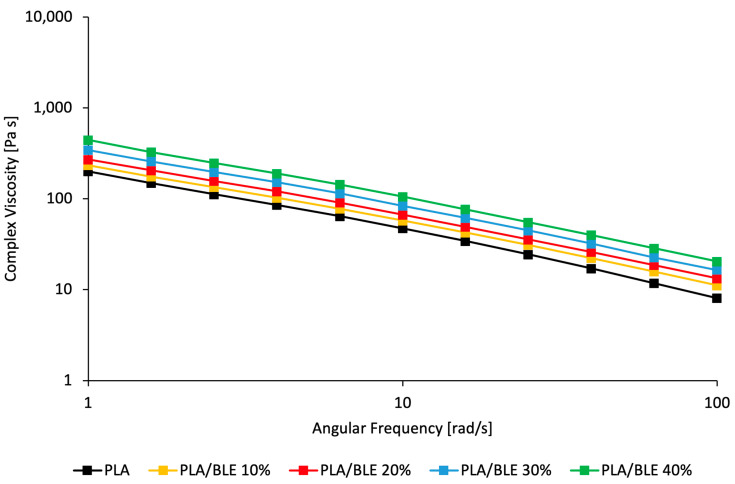
Frequency sweep rheology results on PLA and PLA/BLE electrospun membranes at different concentrations: complex viscosity [Pa s] at varying angular frequency [rad/s] in the range 1–100 rad/s.

The viscosity of polymeric solutions is a key parameter to establish their processability through electrospinning technology. The complex viscosity of the PLA solutions at increasing concentrations of BLE was studied in the range of 1–100 rad/s through a frequency sweep test, reported in Figure 1. The results show that the solution viscosity increases in the presence of BLE compared to pure PLA in the whole range of angular frequency investigated.

In more detail, the viscosity of the suspensions slightly increased with the BLE concentration in the whole range of investigated frequencies. This result was expected since it is well known that solid particles usually increase the viscosity of the polymeric solution, in particular, at high concentrations. However, the increase was found to be relatively low for the high concentrations added to the PLA solutions. This result can be likely explained by taking into account that the polyphenols present in the BLE particles can be released from the filler and facilitate macromolecular sleeping and orientation under applied shear stress [48,49].

Specifically, for the electrospun sample with a concentration of 10 wt% BLE, there is an increase in viscosity with a slight increase in fiber diameter. For the membrane with 20% BLE, the viscosity is slightly higher than pure PLA but lower than 10%, consistent with the fiber diameter size, which was practically unaffected by the presence of BLE. Finally, the fiber diameter of mats with 30% and 40% BLE is smaller than samples containing 10% and 20% BLE despite the viscosity experiencing an increase. For example, at high BLE concentrations, larger quantities of polyphenols released into the polymeric macromolecules are present within the fibers. Polyphenols are well known to affect the macromolecules of polyesters like PLA [49]. Therefore, this phenomenon might cause a reduction in fiber diameters by increasing the mobility of polymeric macromolecules, although the solution viscosity globally increases because of the presence of BLE solid particles [48,50].

### 3.2. Morphology of BLE and Electrospun Mats: Particle Size and Fiber Diameter Distributions

**Figure 2:**
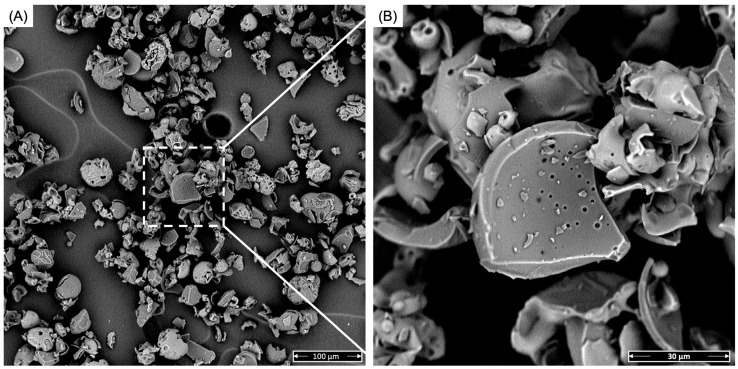
SEM images of the BLE powder: (**A**) micrograph at 100 μm scale bar and (**B**) micrograph at 30 μm scale bar.

Figure 2 shows the morphology of the bamboo leaf extract used for this work. From the figure, it can be observed that the bamboo leaf extract exhibited an irregular shape, mostly spherical, with the presence of fragmented and porous regions. The particle size is within the range of 5–50 μm. These dimensions are in line with SEM analyses performed by Shen et al. [51], which evaluated morphological changes in bamboo leaf extracts following different extraction methods. The particles used in this work seemed smoother and more porous than those used by Shen et al. This porosity may be due to the natural porosity of the bamboo leaves [52].

**Figure 3:**
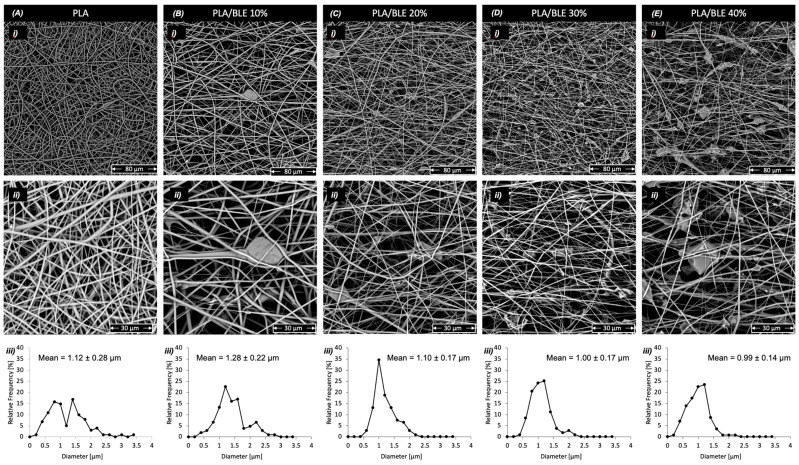
SEM micrographs of electrospun PLA and PLA/BLE electrospun membranes and fiber diameter distribution: (**A**) pure PLA, (**B**) PLA/BLE 10%, (**C**) PLA/BLE 20%, (**D**) PLA/BLE 30%, (**E**) PLA/BLE 40%; (**i**) SEM image with bar scales of 80 microns; (**ii**) SEM image with bar scales of 30 microns; (**iii**) graph of relative frequency [%] of fibers with certain diameter in relation to the fiber diameter [μm]. DiameterJ (v1.018) software was used for graphs (**iii**).

SEM micrographs of the electrospun membranes made with PLA and different concentrations of BLE are shown in Figure 3 as well as the diameter size distribution of the produced fibers. The figures indicate that the material consisting of pure PLA (Figure 3A(i,ii) has the typical morphology of an electrospun membrane as it consists of fibers with diameters on the order of micrometers [53,54]. The fibers appear smooth, randomly oriented, and with a homogeneous diameter distribution of around 1.12 μm (Figure 3A(iii)), in line with previous studies producing PLA electrospun fibers [42].

In PLA/BLE composites, particles of BLE are clearly observable within the fibers, resulting in a rough texture with irregularities that increase in number with higher BLE concentrations (Figure 3B(ii),C(ii),D(ii) and E(ii)). A similar outcome has been observed when using bamboo charcoal in PLA mats produced through electrospinning [55]. Although bamboo charcoal differs from leaf extract, this characteristic is common to both bamboo-based additives.

In addition, with BLE concentrations of 10%, the fiber diameter increases slightly (Figure 3B(iii)). At the 20% concentration of BLE, the fibers appear to have not altered in diameter compared with pure PLA (Figure 3C(iii)). On the contrary, fiber diameter decreases when incorporating BLE concentrations of 30% and 40%, resulting in a smaller diameter size than pure PLA fibers (Figure 3D(iii) and E(iii)).

While an increase in the fiber diameter of PLA/BLE 10% samples was expected, the other results deviated from the conventional trend since, regarding the above-discussed increase in the viscosity of the polymeric solutions with the concentration of BLE, would suggest a monotonical increase in the fiber distribution. In fact, it is well known that, usually, the higher the polymeric solution viscosity, the higher the fiber diameter of electrospun materials [56,57,58].

However, other characteristics of BLE could potentially influence the ability of PLA to be spun during the electrospinning process, leading to a reduction in the fiber size.

In particular, as observed for the viscosity measurements, the lower-than-expected viscosity increase may be connected to the behavior of the polyphenols in the BLE particles, which could ease the PLA macromolecular alignment, facilitating the macromolecular sleeping and orientation during the electrospinning process, resulting in a decrease in the fiber diameter. Polyphenols are well known to increase the macromolecule mobility of polyesters like PLA [49]. Therefore, this phenomenon might cause a reduction in fiber diameters by increasing the mobility of polymeric macromolecules, although the solution viscosity globally increases because of the presence of BLE solid particles [48,50].

### 3.3. Mechanical Behavior

The mechanical behavior of the PLA/BLE electrospun samples was evaluated through the stress–strain curves obtained with tensile tests and compared to that of electrospun PLA.

**Figure 4:**
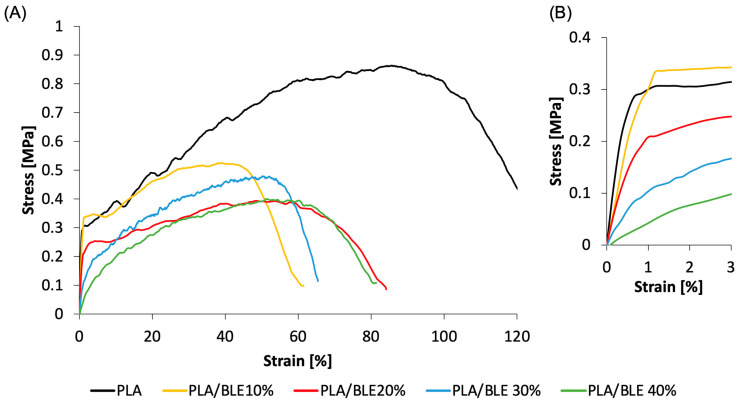
Results of the mechanical tensile test on the electrospun PLA and PLA/BLE specimens: (**A**) stress–strain curve throughout the strain range to failure; (**B**) stress–strain curve in the region of elastic behavior.

As evident from the graph in Figure 4 and Table 1, all tested materials exhibit ductile mechanical behavior with an elastic linear region followed by plastic deformation reaching at least 55%. As observable in the inset of the stress–strain curve (Figure 4B), the slope of the elastic linear region depends on the BLE concentration. Specifically, Table 1 shows that the elastic modulus decreases with increasing BLE concentration, ranging from the highest value of 61 MPa for the pure electrospun PLA to the lowest value of 6.3 MPa for the samples containing 40% BLE. This behavior can be attributed to BLE polyphenol content that can be released from the BLE particles and weaken the interactions among the polymer chains, resulting in a less compact matrix and, consequently, compromising the mechanical strength of the material [1].

Similarly, the maximum stress decreases from 0.87 MPa for the pure PLA membrane to the lowest value of 0.40 MPa for the mats containing 40% BLE. Additionally, the pure PLA electrospun mats exhibit an elongation at a break of 100%, while systems produced with PLA and the addition of BLE show a reduction in the elongation at break to values around 60–70%. To explain this phenomenon, we can consider BLE particles inside the fibers acting as defects, particularly stress concentrators [59]. This effect is evident from the morphology of the fibers containing various concentrations of BLE, which clearly show microscopic cavities and irregularities that can lead to premature fracture of the samples [59]. After analyzing the obtained values, it can be concluded that all materials possess an elastic modulus, maximum stress, and rupture deformation that are compatible with their use in food packaging applications [60,61].

### 3.4. Wettability of Electrospun Mats

An important parameter to be considered in a membrane in contact with food is wettability since it allows for determining the degree of water repellency of the material, i.e., a water resistance property or their ability to absorb exudates, in particular, for porous systems such as electrospun materials.

**Figure 5:**
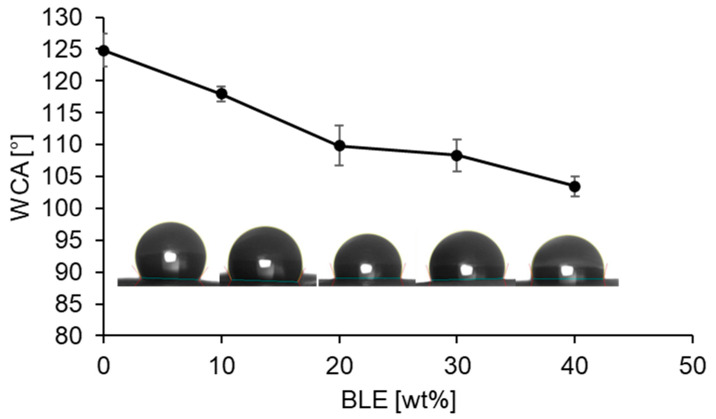
Contact angle [°] at varying BLE content [wt%] obtained from wettability tests.

Figure 5 shows how the trend in water contact angle (WCA) is a function of BLE concentration.

From the results in Figure 5, when the concentration of BLE increases, the contact angle decreases. Specifically, the contact angle for the pure PLA electrospun mat is 125°, consistent with the range of WCA (110–135°) of this material found in the literature [53,62], while for electrospun membranes containing BLE, the contact angle decreases to reach the minimum value within the mat containing 40% BLE (105°). This result can be attributed to the BLE embedding within the fiber, which imparts a certain degree of hydrophilicity since it is a natural compound obtained from a plant. Similarly, Wu et al. [63] studied the wettability of PLA composites reinforced with bamboo fibers, finding that the contact angle decreases with increasing bamboo fiber content in the composite. Therefore, BLE particles, although encompassed and coated by the fibers (Figure 2), impart some hydrophilicity to the membrane, causing a slight increase in wettability, which, however, always remains in the range of hydrophobic material. A decrease in hydrophobicity/hydrophilicity ratio was also observed in electrospun synthetic polymers with added natural extracts in previous studies [64,65]. From a practical point of view, the slight increase in wettability could slightly accelerate the hydrolysis of PLA if used for high-moisture foods. Therefore, this system would be more adaptable for semi-dry foods where no hydrolysis occurred in other PLA-based systems according to scientific literature [66].

### 3.5. Functional Properties of PLA/BLE Electrospun Mats

Active food packaging has become increasingly popular in recent years. These packages not only protect food but also release helpful compounds. They help keep packaged food fresh longer by preventing it from spoiling due to oxidative processes and microbial contamination.

Bamboos are strong, lightweight plants that belong to the grass family Poaceae. Apart from being widely used for construction and craftsmanship due to their lightweight and strength properties, bamboo leaves have been employed for medicinal purposes for a long time [34]. Recent studies have found that bamboo leaf extracts are rich in bioactive compounds, like flavonoids and phenolic acids, which have various health benefits [34,67]. In recent years, bamboo leaf extracts have garnered attention as potent antioxidant additives in animal feed to improve meat quality [32,68]. In addition, they are increasingly being utilized in the food industry for producing additives that enhance flavor or inhibit oxidative processes, thereby extending the shelf life of food products [69]. On the other hand, despite numerous applications of plant extracts to functionalize materials for food packaging, there have been limited applications of bamboo leaf preparations. Some studies have demonstrated that the addition of volatile oil from bamboo leaves imparts antibacterial properties and improves the physicochemical characteristics of corn starch-based films [70]. However, to the best of our knowledge, the effectiveness of bamboo leaf extracts (BLEs) in conferring antioxidant properties to food packaging materials has never been evaluated before.

In order to characterize the commercial bamboo extract used in this study, we measured the TPC and antioxidant activity of BLE using three in-solution standard methods. The TPC values and antioxidant activity, assessed through the DPPH, ABTS, and FRAP assays, are presented in Table 2.

The results show that bamboo leaves contain a high concentration of antioxidant polyphenols, which aligns with previous research [34]. This suggests that adding bamboo leaf extract (BLE) to various products could enhance their antioxidant properties.

To accurately evaluate the antioxidant properties of a putative sample, it is highly recommended to use multiple in-solution assays. Each assay offers unique strengths and limitations that complement one another [71]. When tested with the DPPH assay, the antioxidant activity of BLE was lower compared to the results from the ABTS and FRAP assays. Discrepancies in the hydrophilicity of the reaction mixtures and variations in the effectiveness of BLE’s antioxidant compounds in reducing the different types of reactive species may have influenced the obtained data. For instance, while the DPPH assay is particularly sensitive to hydrogen-donating antioxidants, the ABTS assay can also detect antioxidants through electron-donating mechanisms. On the other hand, the recorded high metal-reducing activity (FRAP value) suggests that BLE components may also exert antioxidant action through chelating actions.

**Figure 6:**
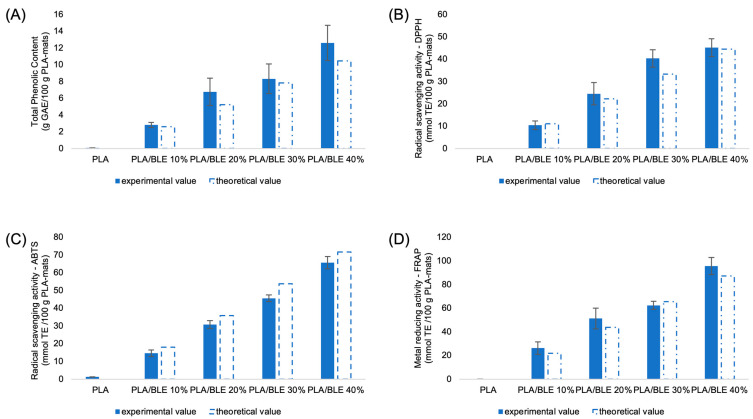
(**A**) Total phenolic content (TPC); (**B**) antioxidant activity assessed by DPPH assay; (**C**) ABTS assay; and (**D**) FRAP assay recovered from PLA-based electrospun samples. Dashed bars represent the expected values estimated based on the TPC, DPPH, ABTS, and FRAP values of BLE. TPC is expressed as mg GAE/100 g membrane. DPPH, ABTS, and FRAP values are expressed as mmol TE/100 g membrane. Values are presented as mean ± SD.

To assess the suitability of BLE for functionalizing PLA-based electrospun membranes, we evaluated its effectiveness when added to the systems at concentrations ranging from 10% to 40% by weight (*w*/*w*). Our goal was to compare the total polyphenol content and the recoverable antioxidant activity of the functionalized electrospun membranes with the values estimated from the polyphenolic content and antioxidant activity of BLE, along with the quantity of functionalizing agent incorporated into the electrospun mat (Figure 6).

TPC values recorded in the functionalized electrospun membranes increased proportionally with the increasing amount of BLE added to the material, reaching a value of 12.61 ± 2.10 g GAE/100 g in the membrane containing 40% BLE. Consistent with the polyphenolic content of the functionalized samples, antioxidant activity increased with the amount of BLE added to the material. Similar to the behavior observed with the functionalizing agent, BLE-infused PLA-based electrospun mats exhibited a higher metal-reducing activity, as evaluated by the FRAP assay, compared to their radical-scavenging activity assessed by DPPH and ABTS assays.

We observed a strong positive correlation among all three assays (FRAP/DPPH, r = 0.9665; FRAP/ABTS, r = 0.9926; ABTS/DPPH, r = 0.9802, *p* ≤ 0.05), as well as a robust correlation between antioxidant activity assays and estimated TPC values (DPPH/TPC, r = 0.9719; ABTS/TPC, r = 0.9950; FRAP/TPC, r = 0.9982, *p* ≤ 0.05).

The antioxidant activity of the PLA-BLE samples increased significantly from the initial value (control membrane) to 45.14 ± 3.99, 65.73 ± 3.49, and 95.64 ± 7.16 mmol TE/100 g membrane with BLE concentrations ranging from 10 to 40 g per 100 g of the membrane, as determined by the DPPH, ABTS, and FRAP assays, respectively.

The close agreement between estimated and experimental values (Figure 6, dashed bars) suggests the stability of the antioxidant components during electrospun material preparation. Additionally, the quantitative recovery of added antioxidant polyphenols indicates the samples’ ability to release the functionalizing agent.

**Figure 7:**
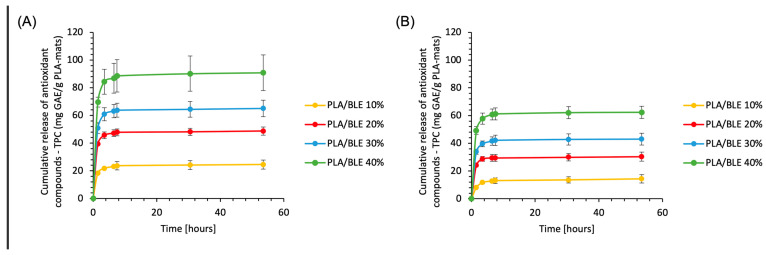
Kinetics of the BLE antioxidant compound released from PLA/BLE electrospun membranes in mixture A (panel **A**) and mixture B (panel **B**). Measurement conditions were as reported in Methods. Data are the mean ± SD of three separate experiments carried out in triplicate.

Considering the high correlation between data from antioxidant activity assays and the Folin–Ciocalteu method, to explore the release behavior of antioxidant components from BLE-infused PLA-based electrospun membranes, we utilized the Folin–Ciocalteu assay to measure the total polyphenolic content (TPC). The samples were subjected to release studies using two distinct food simulants, representing aqueous and lipid-based matrices, to examine their influence on antioxidant release. Figure 7 illustrates the release profiles of BLE antioxidants from electrospun membranes functionalized with concentrations ranging from 10% to 40% by weight.

The results reveal that variations in membrane hydration levels, influenced by the food simulant’s polarity and the relative affinity of antioxidant compounds with PLA and the release medium, impact the extent of antioxidant release. As depicted in Figure 2, the accumulation of antioxidants in the release media follows an exponential kinetic until reaching a maximum value.

Generally, the cumulative TPC values measured in the food simulants at the test’s conclusion were moderate compared to those achievable from each electrospun mat, 77% for mixture A and 49% for mixture B. The differences in release between the two simulants can be attributed to the moderately lipophilic nature of many polyphenolic flavonoids, such as those abundant in bamboo extract.

Despite studies suggesting that certain phytochemicals, like catechins, may act as crosslinking additives, thereby reducing release from the material, our findings demonstrate elevated releases for PLA/BLE electrospun mats, especially in simulant A [72,73]. This suggests a high propensity of the polymeric matrix to release antioxidant components. The significant capacity of the samples to release BLE active compounds is further evidenced by their rapid release in both simulants, with the TPC value at 3.5 h averaging 90% of the total cumulative release.

It is important to emphasize that the experimental conditions used to evaluate the antioxidant release from the PLA-BLE membrane were conducted under ‘sink conditions’, where the food simulant was completely replaced at set intervals. This method maintains a high concentration gradient between the materials and the simulant, avoiding saturation in the simulant and ensuring a consistent release of antioxidants from the electrospun membrane. These conditions represent a deliberately accelerated and stressed release scenario, designed to measure the membrane’s maximum potential release capacity for the antioxidant.

In real-life applications, true sink conditions would not typically be achieved, meaning that the release of the antioxidant would likely occur more slowly and steadily over an extended period.

## 4. Conclusions

This study developed and investigated active electrospun mats with antioxidant properties using electrospinning technology. Electrospinning enabled the incorporation of high concentrations of natural antioxidant compounds (up to 40%), potentially applicable in the food packaging field. Micrometric fibers were obtained, allowing for the controlled release of antioxidant compounds. Overall, the presence of BLE slightly influenced the fiber diameter and increased the viscosity of the polymer solution for electrospinning. Mechanical and wettability tests demonstrated that adding BLE decreased the modulus, maximum stress, and strain at break and increased the hydrophilicity compared to pure PLA proportionally to the embedded concentration. However, this property alteration has maintained the hydrophobic behavior of the material and, overall, does not hinder potential use in the packaging industry. Total polyphenol content, estimated through FOLIN assay, increased proportionally with incorporated BLE, impacting antioxidant properties assessed via FRAP assay.

## Figures and Tables

**Table 1:** Elastic modulus (E), maximum stress (TS), and strain at break (ε_b_) of the produced electrospun membranes.

	E [MPa]	TS [MPa]	ε_b_ [%]
PLA	60.89 ± 4.26	0.87 ± 0.08	100.8 ± 7.1
PLA/BLE 10%	40.08 ± 2.12	0.58 ± 0.04	55.7 ± 5.0
PLA/BLE 20%	20.86 ± 1.67	0.47 ± 0.02	67.8 ± 6.7
PLA/BLE 30%	10.50 ± 0.84	0.41 ± 0.05	59.0 ± 2.4
PLA/BLE 40%	6.31 ± 0.41	0.40 ± 0.05	69.2 ± 4.8

**Table 2:** Total polyphenolic content and antioxidant activity of bamboo leaf extract. Values are expressed as mean ± SD of three experiments carried out in triplicate.

TPC	24.71 ± 2.62	g GAE per 100 g of DW
DPPH	111.10 ± 6.46	mmol TE per 100 g of DW
ABTS	179.26 ± 20.34	mmol TE per 100 g of DW
FRAP	218.37 ± 16.47	mmol TE per 100 g of DW

## Data Availability

The data presented in this study are available on request from the corresponding author.
